# Plasma hyperosmolality during cardiopulmonary bypass is a risk factor for postoperative acute kidney injury: Results from double blind randomised controlled trial

**DOI:** 10.1177/02676591241240726

**Published:** 2024-03-21

**Authors:** Staffan Svenmarker, Helena Claesson Lingehall, Gunnar Malmqvist, Micael Appelblad

**Affiliations:** 1 Department of Public Health and Clinical Medicine, 174459 Umeå University, Umeå, Sweden; 2 Department of Nursing, 8075 Umeå University, Umeå, Sweden; 3 Department of Public Health and Clinical Medicine, Heart Centre, 174459 Umeå University, Umeå, Sweden

**Keywords:** cardiac surgery, acute kidney injury, cardiopulmonary bypass, osmolar concentration, heart-lung machine, priming solution

## Abstract

**Introduction:**

The study objective was to investigate whether a Ringer’s acetate based priming solution with addition of Mannitol and sodium concentrate increases the risk of cardiac surgery associated kidney injury (CSA-AKI).

**Methods:**

This is a double blind, prospective randomized controlled trial from a single tertiary teaching hospital in Sweden including patients aged ≥65 years (*n* = 195) admitted for routine cardiac surgery with cardiopulmonary bypass. Patients in the study group received Ringer’s acetate 1000 mL + 400 mL Mannitol (60 g) + sodium chloride 40 mL (160 mmol) and heparin 2 mL (10 000 IU) 966 mOsmol (*n* = 98), while patients in the control group received Ringer’s acetate 1400 mL + heparin 2 mL (10 000 IU), 388 mOsmol (*n* = 97) as pump prime. Acute kidney injury was analysed based on the Kidney Disease Improving Outcomes (KDIGO 1-3) definition.

**Results:**

The overall incidence of CSA-AKI (KDIGO stage 1) was 2.6% on day 1 in the ICU and 5.6% on day 3, postoperatively. The serum creatinine level did not show any postoperative intergroup differences, when compared to baseline preoperative values. Six patients in the Ringer and five patients in the Mannitol group developed CSA-AKI (KDIGO 1-3), all with glomerular filtration rates <60 mL/min/1.73 m^2^. These patients showed significantly higher plasma osmolality levels compared to preoperative values. Hyperosmolality together with patient age and the duration of the surgery were independent risk factors for postoperative acute kidney injury (KDIGO 1-3).

**Conclusions:**

The use of a hyperosmolar prime solution did not increase the incidence of postoperative CSA-AKI in this study, while high plasma osmolality alone increased the associated risk by 30%. The data suggests further examination of plasma hyperosmolality as a relative risk factor of CSA-AKI.

## Introduction

The incidence of cardiac surgery associated acute kidney injury (CSA-AKI) varies between 8.9%–50%.^1,2^ It has been reported as an independent risk factor for mortality, with odds ratio (OR) of 5.3.^3^ The ethology of CSA-AKI is multifactorial.^1,2,4^ One possible risk factor is the priming solution used for cardiopulmonary bypass (CPB). Its composition varies extensively between cardiac units and nations.^5^ Existing guidelines are inconclusive,^6^ which to some extent may explain the observed variability. The typical prime contains a balanced crystalloid solution combined with a synthetic or albumin colloid.^5^ What would represent an ideal mix of components still remains an open question mainly depending on the lack of high quality studies.^7^

Mannitol is an osmotic diuretic widely used as prime additive in combination with a crystalloid component.^1,8^ It is generally reported to be a safe alternative,^1^ however, with disputable effects on renal function.^1,9,10^ Dosing effects of Mannitol should also be considered, which make outcome measures difficult to assess.^11^ Large doses of Mannitol will markedly increase the osmolality of the priming solution, when combined with electrolyte additives.^12^

The present randomised controlled trial aims to investigate whether a Ringer’s acetate based priming solution with addition of Mannitol and sodium chloride increases the risk for CSA-AKI.

## Methods

### Study design

This prospective double-blind single-centre randomised controlled trial refers to a previous publication (EudraCT-nr 2018-002,385-39).^13^ The present ad hoc protocol was approved by the Swedish Ethical Review Authority (Dnr 2023-02,241-02) and registered in Clinical Trials.gov (NCT05914896). In brief, the study cohort included patients aged >65 years scheduled for cardiac surgery at the Heart Centre of Umeå University Hospital, Sweden. Patients requiring acute surgery within 24 h or profound hypothermia or with previously documented allergic reactions were not included.

Randomisation (1:1) was established from a list of predefined computer-generated random values. Results of randomisation was disguised for both patients and treating personnel. Patients randomised to the Mannitol group (966 mOsm) received Ringer’s acetate 1000 mL + 400 mL Mannitol (60 g) + sodium chloride 40 mL (160 mmol) and heparin 2 mL (10 000 IU), while patients in the Ringer group (388 mOsm) received Ringer’s acetate 1400 mL + heparin 2 mL (10 000 IU) as pump prime.

### Cardiopulmonary bypass and general anaesthesia

The systemic blood flow control aimed to preserve mean arterial pressure (MAP) > 50 mmHg and mixed venous oxygen saturation (S_v_O_2_) >75% by adjustment of roller pump settings or by adding norepinephrine or phenylephrine. The target body temperature was 34°C. Heparin was titrated to elevate the activated clotting time >480 s. Shed blood was retransfused continuously. The general anaesthesia comprised a combination of propofol (5–10 mg/kg), fentanyl (1–7 μg/kg), rocuronium (0.6–1.0 mg) and sevoflurane (1 MAC).

### Staging of acute kidney injury

Staging of acute kidney injury (AKI) followed the Kidney Disease Improving Outcomes (KDIGO) definition.^14^ Stage I defines a relative baseline increase of the serum creatinine level by ≥ 1.5 1.9, stage II: ≥2 2.9 and stage III: ≥3.0. Staging by the estimated glomerular filtration rate (eGFR 1.73 m^2^) was also implemented using the 3A level equal to <60 mL/min/1.73 m^2^. This defines a patient with mildly to moderately reduced renal function.

### Laboratory data

The results for standard laboratory chemistry tests were collected from hospital registries. Plasma osmolality analyses were added for the purpose of this study. Sampling occurred preoperatively, before CPB, after CPB, in the intensive care unit (ICU) after 12 h and on the 3^rd^ day postoperatively.

### Statistical methods

Continuous variables were evaluated using Q-Q plots and histograms to determine its distribution characteristics, including the Shapiro-Wilk’s test of normality. Variables fulfilling the requirement for a normal distribution were analysed using the *t* test; elsewise, the Mann-Whitney u-test to statistically verify intergroup differences. Categorical variables were analysed in contingency tables using the Pearson’s chi-squared test, alternatively Fischer’s exact test or by reporting the linear association value as indicated. Risk analysis was performed by logistic regression modelling and reported as odds ratios (OR) with its associated 95% confidence interval (95% CI). A *p*-value less than 0.05 was regarded as statistically significant. The SPSS software version 28 (IBM corporation, Armonk, New York, USA) was used for all analyses.

## Results

### Background information

**Figure 1.**
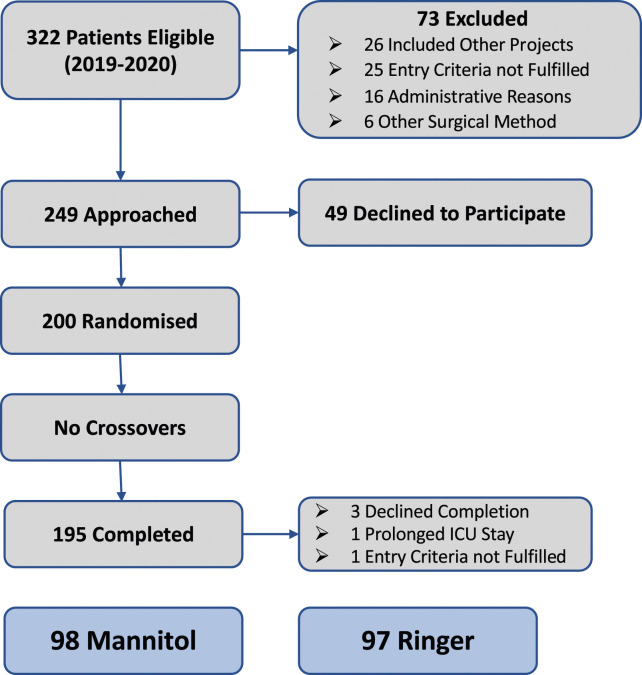
Schematic illustration of the enrolment process. Detailed outlined in reference no 13.

Of the 200 randomised patients, 195 completed the intension to treat study design as specified in Figure 1. Ninety-eight (*n* = 98) patients formed the Mannitol group and (*n* = 97) the Ringer group. The background characteristics for the two groups are described in Tables 1–3. Post intervention showed few statistically verified intergroup differences. However, the intraoperative volume balance was predominantly more favourable (*p* < 0.001) among Mannitol patients based on higher urine output (*p* < 0.001) and in addition to a lower haematocrit level (*p* < 0.001) during CPB. The potassium and sodium levels showed small, but statistically significant intergroup differences postoperatively (Table 3). No patient required renal replacement therapy.

**Table 1.** Specification of the patient’s background characteristics.

	All (*n* = 195)	Mannitol (*n* = 98)	Ringer (*n* = 97)
Age (years)	73 ± 4.4	73 ± 4.3	73 ± 4.6
Female sex (%)	27	30	25
BMI (kg/m^2^)	27 (5)	27.0 (5)	27.0 (5)
Hypertension (%)	82	85	78
Diabetes (%)	22	22	22
COPD (%)	4	4	3
EuroSCORE II	1.9 (2)	1.7 (1.7)	2.0 (2.1)
Left Ventricular function (%)			
Mildly decompensated	17	18	17
Moderately decompensated	11	10	12
Severely decompensated	2	0	4
Atrial fibrillation (%)			
Paroxysmal	10	10	10
Permanent	6	7	4
Persistent	4	5	2
Smoker (%)	4	6	2
Alcohol use (%)	67	68	66

Alcohol > once a month. Smoker >20 cigarettes per year. COPD, Chronic Obstructive Pulmonary Disease. Diabetes summary of types 1 and 2. Central tendency means ± SD or median (IQR). EuroSCORE II, The European System for Cardiac Operative Risk Evaluation.

**Table 2.** Perioperative patient related observations.

	All (*n* = 195)	Mannitol (*n* = 98)	Ringer (97)	*p*-value
CPB duration (min)	89 (42)	87 (43)	92 (44)	0.22
Aorta clamped (min)	62 (35)	60 (33)	66 (38)	0.37
Surgery duration (min)	177 (60)	173 (64)	178 (58)	0.25
Mean arterial pressure (mmHg)	63 (7)	63 (7)	63 (8)	0.52
Mixed venous saturation (%)	80 (5)	80 (5)	79 (5)	0.83
Haematocrit CPB (%)	31 (6)	30.2 ± 4.0	32.1 ± 3.7	<0.001
Volume balance intraoperatively (ml)	2120 (1345)	1917 (1491)	2354 (1203)	<0.001
Urine output intraoperatively	420 (390)	573 (573)	330 (245)	<0.001
Chest tube drainage ICU 24 h (ml)	640 (430)	640 (495)	650 (410)	0.85
Blood transfusion (%)	41	43	40	0.60
Red cell transfusion				0.63
1-2 units (%)	25	27	24	
3-5 units (%)	10	10	10	
>6 units (%)	4	4	3	
Plasma transfusion				0.28
1-2 units (%)	8	9	4	
3-5 units (%)	3	4	2	
>6 units (%)	2	2	2	
Platelet transfusion				0.59
1-2 units (%)		9.2	6.2	
3-5 units (%)		2.0	2.1	
ICU stay (Hr)	22 (6)	23 (6)	22 (4)	0.21
Ventilator duration ICU (h)	6.5 (3)	6.6 (3)	6.5 (3)	0.34
30-days mortality	0	0	0	-

Central tendency means ± SD or median (IQR). A *p*-value <0.05 was considered statistically significant.

**Table 3.** Laboratory chemistry overview.

	All (*n* = 195)	Mannitol (*n* = 98)	Ringer (*n* = 97)	*p*-value
Creatinine (μmol/L)				
Baseline	82 (25)	83 (28)	81 (22)	0.36
Day 1	76 (38)	76 (32)	78 (23)	0.48
Day 3	77 (27)	79 (34)	76 (26)	0.62
eGRF (ml/min/1.73 m^2^)				
Baseline	77 (22)	75 (26)	80 (19)	0.20
Day 1	82 (24)	80 (26)	83 (15)	0.28
Day 3	83 (27)	81 (30)	86 (26)	0.26
Plasma osmolality (mOsm/kg)				
Baseline	295 (7)	295 (6)	294 (7)	0.29
Commence CPB	305 (28)	323 (14)	295 (5)	<0.001
After CPB	302 (12)	309 (8)	297 (5)	<0.001
Day 1	290 (9)	294 (9)	288 (7)	<0.001
Day 3	290 (7)	291 (8)	289 (7)	0.35
Potassium (mmol/L)				
Baseline	4.1 (0.4)	4.1 (0.5)	4.1 (0.4)	0.80
Day 1	4.4 (0.3)	4.4 (0.4)	4.5 (0.4)	0.08
Day 3	4.1 (0.4)	4.1 (0.4)	4.2 (0.4)	0.02
Sodium (mmol/L)				
Baseline	140 (2)	140 (3)	140 (2)	0.22
Day 1	138 (4)	139 (3)	137 (3)	<0.001
Day 3	137 (3)	137 (5)	137 (3)	0.61
Haemoglobin (g/L)				
Baseline	135 ± 14	136 ± 14	135 ± 14	0.78
Day 1	101 (17)	101 (16)	100 (16)	0.96
Day 3	92 (16)	93 (15)	90 (15)	0.26
Leucocytes (10^9^/L)				
Baseline	6.9 (2.1)	6.8 (2.3)	7.1 (2.0)	0.36
Day 1	11 (4.2)	11 (4.5)	11 (3.7)	0.22
Day3	9.7 (3)	9.6 (4.0)	9.7 (2.9)	0.74
Platelets (10^9^/L				
Baseline	226 (68)	220 (67)	230 (67)	0.76
Day 1	155 (51)	154 (57)	156 (54)	0.53
Day3	141 (61)	135 (60)	143 (63)	0.38
Baseline values				
B-type natriuretic peptide (ng/L)		561 (1127)	351 (815)	0.49
C-Reactive protein (mg/L)		1.3 (2.7)	1.5 (3.4)	0.28

eGFR: Estimated glomerular filtration rate. Central tendency: median (IQR) or mean ± standard deviation. A *p*-value <0.05 was considered statistically significant.

### Hyperosmolality and acute kidney injury

**Figure 2.**
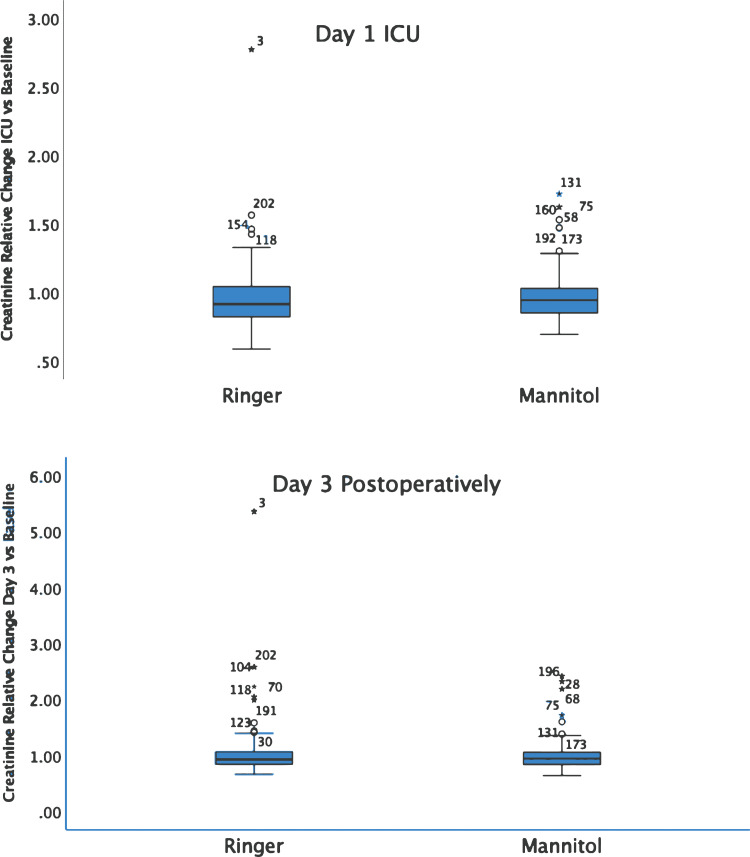
The relative change of the creatinine level versus baseline measured in the intensive care unit day 1 (upper panel) and on day 3 postoperatively (lower panel). Intergroup differences were statistically insignificant (*p* > 0.05) on both occasions. Outliers indicated by patient notations.

The increase of the serum creatinine level compared to baseline at different time points did not show any intergroup differences. The level was 0.97 (0.18) vs. 0.91 (0.22) (*p* = 0.47) in the ICU and 0.94 (0.22) vs. 0.93 (0.23) (*p* = 0.98) on day 3 postoperatively (Figure 2). 11 patients were identified with postoperative eGFR values less than 60 mL/min/1.73. These patients demonstrated significantly higher osmolality levels throughout the perioperative phase compared to patients with normal eGFR: prior to surgery (295 (6) vs. 294 (6) mOsm/kg, *p* = 0.01), at the end of CPB (305 (12) vs. 302 (12) mOsm/kg, *p* = 0.04), ICU (291 (8) vs. 289 (10), *p* = 0.01) and on day 3 postoperatively: 293 (10) versus 289 (7) mOsm/kg (*p* < 0.001).

The incidence of AKI (KDIGO stage 1) was 2.6% on day 1 in the ICU and 5.6% on day 3 postoperatively. The intergroup incidence is displayed in Table 4. The risk for developing postoperative AKI did not differ between Mannitol and Ringer allocated patients. Associated OR was 1.2 (95% CI 0.58 – 2.49) in the ICU and 0.90 (95% CI 0.46 – 1.75) on the 3^rd^ post2operative day.

**Table 4.** Incidence of postoperative acute kidney injury.

KDIGO stratification	Mannitol (*n* = 98)	Ringer (*n* = 97)	*p*-value
Acute kidney injury ICU day 1			0.99
Stage 1 (%)	3.1	1.0	
Stage 2 (%)	0	1.0	
Stage ≥1 (%)	3.1	2.1	1.00
Acute kidney injury day 3			0.71
Stage 1 (%)	2.0	1.0	
Stage 2 (%)	3.1	3.1	
Stage 3 (%)	0	1.0	
Stage ≥1 (%)	5.1	6.1	0.74

Staging of acute kidney injury according to the KDIGO definition.^14^ A *p*-value <0.05 was considered statistically significant.

### Risk factors associated with acute kidney injury

11 patients (*n* = 11) were diagnosed with AKI (KDIGO 1-3) on the 3^rd^ day after surgery: five patients in the Mannitol group and six patients in the Ringer group; the measured eGFR was 31 (8) ml/min/1.73 m^2^. Based on logistic regression analysis, the following covariates were identified as independent risk factors: patient age, duration of surgery and plasma osmolality measured at the same time point. Table 5 gives a detailed overview.

**Table 5.** Risk factors for acute kidney injury 3 days after surgery.

Variables	Model coefficient	p-value	Odds Ratio	95% CI
Patient age	0.28	0.007	1.32	1.14–1.45
Plasma osmolality day 3	0.27	<0.001	1.31	1.14–1.51
Duration surgery	0.02	0.04	1.02	1.001–1.04

Logistic regression model using a forward stepwise (Wald) procedure with inclusion of the following covariates: Sex, Patient age, Diabetes mellitus, EuroSCORE II, Urine output during surgery, Haemoglobin 3^rd^ day postoperatively, Hypertension, Atrial fibrillation, Duration cardiopulmonary bypass, Duration surgery, Mixed venous oxygen saturation during cardiopulmonary bypass, Haematocrit during cardiopulmonary bypass, Mean arterial pressure during cardiopulmonary bypass, Blood component therapy, Creatinine baseline, Haemoglobin baseline, Plasma osmolality at commence of cardiopulmonary bypass, Plasma osmolality at weaning from cardiopulmonary bypass, Plasma osmolality 1^st^ day postoperatively, Urine output 12 h. Model summary: 0.48 (Nagelkerke R Square). A *p*-value <0.05 was considered statistically significant.

## Discussion

The results from this study suggest that Mannitol combined with sodium chloride as pump prime additives is safe. The postoperative CSA-AKI incidence remained the same compared to patients receiving a clear Ringer based prime. However, notable is that the risk for postoperative CSA-AKI increased by 30% in a selection of hyperosmolar patients. Other independent risk factors for CSA-AKI were old age and the duration of surgery. The identified risk, relative hyperosmolality warrants further investigations.

A retrospective analysis of renal function over a 10-year period from our unit included a wide spectrum of cardiac surgical procedures (*n* = 6945) and confirmed the safety profile of the Mannitol prime additive.^15^ The CSA-AKI incidence in that study ranged between 7.5 and 2.6%–0.6% based on the AKIN I-II-III criterion. These results were very similar to the incidence reported for the Mannitol group in the present randomised trial. The incidence of AKI after cardiac surgery varies considerably 5%–40% as shown by Milne et al.^4^ This suggests a multifactorial origin of AKI, where the influence of different prime compositions probably was less relevant. For instance, Kolsrud at al^16^ reported the AKI incidence of 18% for the dextran based prime solution PrimeECC^®^ (XVIVO Perfusion) versus 22 % for the Ringer prime alternative. These major CSA-AKI incidence differences reported in varied investigations cannot solely be explained by the pump prime composition itself. Other risk factors to consider are adverse effects related to CPB there several physiological reactions are at play as for example an immediate 20% reduction of the renal blood flow at the start of the CPB induced by renal vasoconstriction^9^ following the renin-angiotensin system activation.^17^ A simultaneous haemodilution reduces the renal oxygen delivery, which is compensated by increasing renal oxygen extraction to maintain the balance between delivery and demand. This abnormal physiological state will continue over the ICU stay increasing the risk for CSA-AKI even more.^9^ It should also be emphasised that even short periods of renal hypoxia may influence renal function since only 10% of the renal blood flow supports the medulla region but is responsible for about 90% of the renal oxygen extraction.^17^ It explains why a sufficient oxygen delivery is paramount both during^4,18–20^ and after CPB.^9,21^

Mannitol is an osmotic diuretic commonly used in cardiac surgery to enhance urine output and facilitate fluid balance control.^22^ It is freely filtered by the glomeruli,^23^ while 7% is reabsorbed.^24^ Bragadottir et al demonstrated in a group of cardiac surgical patients with postoperative AKI how Mannitol increases urine output accompanied by an increase of the renal blood flow and a lower renal vascular resistance without disturbing either renal filtration fraction or oxygenation.^10^ Despite all Mannitol attributed favourable and non-favourable effects^10,17,25–27^ there is no convincing evidence that Mannitol can be used to protect against the development of AKI after cardiac surgery.^4,17,26^ In this study we used hypertonic saline combined with Mannitol as CPB prime additives. The concept of using hypertonic saline during CPB is sparsely described. However, Järvelä et al reported reduced fluid accumulation when hypertonic saline was combined with hydroxyethyl starch.^28^

The examined prime solution is safe from a general perspective.^15^ Nevertheless, we cannot ignore the somewhat disturbing alternative finding that hyperosmolality per se seems to increase the risk of postoperative AKI. Malmqvist et al demonstrated how the investigated Mannitol prime increased the plasma osmolality well above 320 mOsm/kg at the start of the CPB but returned to a normal level during the ICU stay.^12^

11 patients developed AKI (KDIGO≥1) 3 days after surgery in the present study, with an even distribution in the two groups equal to an overall incidence of 5.6%. The fact that hyperosmolality is associated with a higher risk of AKI is demonstrated by several investigations. Farhan et al conducted a retrospective analysis of *n* = 1927 percutaneous coronary interventions.^29^ The infusion of hyperosmolar dye increased the risk for both postoperative AKI and the 1-year mortality. Similar results were presented in a retrospective cohort analysis of more than 20-thousand critically ill patients.^30^ The risk of developing AKI in the ICU increased by 33% in patients with serum osmolality exceeding 300 mOsm/kg. Their findings concur closely with our own results, except for the incidence difference (5.6 vs 38%). Seeliger et al outlined possible reasons why hyperosmolality may destabilise normal renal function.^31^ Hyperosmolar injury can only occur if the tubular fluid exceeds the encircling medullary osmolality, which theoretically seems possible for a hyperosmolar prime exceeding 900 mOsm/kg. Direct osmotic nephrosis of tubular cells has also been postulated as a mechanism^11,32^ or alternatively pinocytosis.

The culprit mechanisms behind CSA-AKI are not fully understood. One risk factor to consider is preoperative hyperosmolar dehydration (HD), especially common among older hospitalised patients. In a recent study, 6632 patients admitted to a large UK university teaching hospital were followed, 27% fulfilled criteria for HD and experienced a fourfold increased risk of developing postoperative acute kidney injury and a 60% greater 30-days mortality compared to euhydrated patients.^33^ Recent guidelines recommend preoperative rehydration by permitting the patient to drink clear liquids up until 2 h prior to induction of general anaesthesia.^2^ Notable is that patients in this cohort arrived in the operating room with osmolality values close to the upper reference limit. To balance preoperative fluid intake is indeed challenging, since overhydration also is associated with serious complications.

Hyperosmolar changes of the circulating blood may also distress normal cerebral functions. Postoperative delirium (POD) is commonly occurring after cardiac surgery with a reported incidence at 25%–46%.^34–36^ One possible risk factor for POD is linked to the selection of the prime solution used for CPB. Recent findings from our group confirm that this may be the case, even if we have been unable to verify this by changes in the postoperative incidence of postoperative delirium.^13^ However, hyperosmolality per se was associated with POD. Further risk analyses are therefore warranted to delineate the influence of hyperosmolality in relation to postoperative delirium and acute kidney injury.

## Conclusion

The use of a hyperosmolar prime solution did not increase the incidence of postoperative CSA-AKI in this study, while high plasma osmolality per se increased the associated risk by 30%.

## Limitations

A limitation to this study is that it is a single centre study, only including patients above 65 years. This limits the generalisation of the findings. Since prime composition is heterogenic among centres no relevant study based on the same prime composition is available for comparison.
